# BACKSLIDING ON CHILDHOOD IMMUNIZATIONS DUE TO ONGOING COVID-19 PANDEMIC: A RETROSPECTIVE STUDY IN BANADIR REGION, SOMALIA

**DOI:** 10.21010/Ajidv17i2S.2

**Published:** 2023-08-01

**Authors:** OREY, Fartun Abdullahi H, SHEIK MOHAMUD, Kadra Hassan, ABDULLE Iftin Abdi Nor, MOHAMOUD, Jamal Hassan, GARBA, Bashiru, ADAM, Mohamed Hussein, DAHIE, Hassan Abdullahi, SH. NUR, Maryan Abdullahi, DIRIE, Najib Isse

**Affiliations:** 1 Department of Pediatrics and Child Health, Dr Sumait Hospital, Faculty of Medicine and Health Sciences, SIMAD University, Mogadishu, Somalia; 2 Faculty of Medicine and Health Sciences, SIMAD University, Mogadishu, Somalia; 3 Department of Public Health, Faculty of Medicine and Health Sciences, SIMAD University, Mogadishu, Somalia; 4 Nursing and Midwifery Department, Faculty of Medicine and Health Sciences, SIMAD University, Mogadishu 2526, Somalia; 5 Department of Obstetrics and Gynecology, Dr Sumait Hospital, Faculty of Medicine and Health Sciences, SIMAD University, Mogadishu, Somalia; 6 Department of Urology, Dr. Sumait Hospital, Faculty of Medicine, and Health Sciences, SIMAD University, Mogadishu, Somalia; 7 Department of Veterinary Public Health and Preventive Medicine, Faculty of Veterinary Medicine, Usmanu Danfodiyo University, Sokoto. 840212, Sultan Abubakar Road, City Campus Complex, Sokoto State, Nigeria

**Keywords:** SARS-CoV-2 virus infection, COVID-19 pandemic, routine childhood immunization, Polio, Pentavalent vaccine, vaccination coverage,

## Abstract

**Background:**

SARS-CoV-2 has resulted in a global public health crisis. During the pandemic, considerable delay was observed making it impossible for some children to receive their due vaccines on time. Like most resource-poor countries, COVID-19 pandemic is thought to have a negative impact on Somalia’s immunization coverage.

**Materials and methods:**

This study aimed to assess the impact of the COVID-19 pandemic on routine childhood immunization coverage in Somalia. A retrospective comparative cross-sectional approach was employed to investigate the number of under-5-year children who got their immunization from the two major mother and child hospital, (Banadir and SOS hospitals) in Mogadishu, Somalia from October 2019 to December 2020. To do this, a total of 112, 060 data relating to the routine childhood immunization (measles, polio, whooping cough, hepatitis B, pneumonia, and tuberculosis) were collected from the monthly immunization report-data from the two hospitals.

**Results:**

The results showed that all the vaccines except birth vaccines have remarkably dropped with Penta-3 (27%), Penta-2 (11%), measles (10%) and Penta-1 (8%) respectively. However, the birth vaccines (BCG and Polio 0) were not affected as observed in this study. The reduction in children immunization rate in Somalia may be a combination of many other factors, we however recognize that the COVID-19 pandemic may have contributed significantly to this outcome .

**Conclusion:**

The government needed to take proactive measures to encourage parents to present their children for immunizations, including increasing community awareness concerning the importance of these routine childhood immunizations despite the ongoing COVID-19 pandemics.

## Introduction

The trail of devastation following the emergence of the SAR-CoV-2 virus outbreak ranging from global and national economy, public health and healthcare service delivery, and death has been unprecedented. The impact especially on health has become even more significant following government’s effort to regulate and stem the further spread of the infection by implementing nation-wide and regional lockdowns, including closure of borders and restriction of movement as well as halting mass gatherings (Garba *et al.*, 2020; Lassi *et al.*, 2021). It was only at the end of the initial lockdown, that the magnitude of the disruptions was realized. In addition to making life extremely difficult and unbearable for families who depend on daily subsistence to feed their families, it became obvious that these restrictions had to be suspended because of the effect it has had on many public health intervention efforts including routine childhood immunization (Oyo-Ita *et al.*, 2016; Khatiwada *et al.*, 2021).

According to the World Health Organisation WHO, a steady increase in the number of children who missed out on the basic vaccines was noted (from 3.7 million in 2019 to 23 million children in 2020). Their findings also indicate that majority of the countries are also recording considerable drops in the childhood immunization rates, further exacerbating the already widening inequalities in vaccine access, with the most affected being children from countries with poor healthcare services and those affected by conflict, wars and natural disasters like drought and famine (WHO 2020). In Africa, the COVID-19 pandemic has spread over African contents leading to sufferings consistent with the global situation across all facets of the society’s health, security, political, economic and social impacts. The fragile situation is becoming worse with mortality due to COVID-19 day after day, unfortunately, most patients don’t get enough oxygen from hospitals (Abbas et al., 2020). The government ordered lockdown and distance in space to minimize the disease outbreak, the essential health services have also been disrupted in many African countries resulting in an imbalance of the demand and supply factors (Abbas et al., 2020).

In Somalia, children receive immunizations against polio, diphtheria. tetanus, pertussis, hepatitis B, pneumonia, measles, and tuberculosis, before their first birthday. The immunizations are free and equally accessible (UNICEF, 2021). However, Somalia is one of the countries with the lowest immunization coverage in Africa even before the outbreak of the SARS-CoV-2 virus infection (Hayir et al., 2020). The low immunization coverage is believed to have multifactorial causes in Somalia. Somalia is surviving decades of civil war, which lead to the fragility and instability of the country’s health system. This has resulted in increased incidence of infectious diseases with high cases of mortality (Moussa et al., 2021; Abdullahi et al., 2022; Aden and Bashiru, 2022; Dahie et al., 2022; Nur et al., 2022). Since the pandemic began, a considerable decline in the number of primary healthcare visits and childhood immunization has also been noted, with parents expressing fear to present their children for routine vaccination due to the possibility of contracting the disease (Coker et al., 2021). This is especially a justified apprehension because most of the war-ravaged countries have difficulty observing the required preventive measures against the COVID-19 such as physical distancing and handwashing, disinfection as well as wearing a mask (ReliefWeb, 2022). Similarly, the WHO reported that 80 million children from 8 countries around the world are at risk of immunizable diseases due to quarantine, isolation, and social distancing. The lockdown and suspension of other healthcare services including immunization campaigns in addition to channeling of all resources to the fight against the dreaded virus may lead Somalia’s children suffering immunizable diseases such as measles. Moreover, the burden seems to increase because most of the parents are compelled to practice the social distance and avoid public gatherings (Ahmed et al., 2020; Adam et al., 2022). On the other hand, many parents have expressed worry over the possibility of contracting COVID-19 from visiting health facilities, hence many parents are refusing to take their children for immunization.In this study, we focused on finding out the impact of the COVID-19 pandemic on routine childhood immunization coverage in Somalia. Knowing childhood immunization coverage during this pandemic may help the informed decision on advocacy to increase the rate of immunization coverage for the benefit of the children and their parents. In addition, the results from this study can be used by relevant health authorities and the Somali government to increase public awareness regarding immunization coverage during the COVID-19 pandemic. Also, it will be useful for future researchers as it will serve as a guide for them to follow up on future studies related to the problem being investigated as well as literature sources for future studies.

## Materials and Methods

### Study design

We conducted a retrospective comparative cross-sectional study targeting children under 5 years who take their immunization from Banadir and SOS (Save Our Souls) hospitals in Mogadishu. The Banadir Hospital is a Women and Children’s Hospital in Mogadishu, Wadajir district. It was built in 1977 as part of the Chinese government development projects to help Somali people. The hospital is a 700 beds capacity hospital that is divided into three main departments namely, Pediatrics, Maternity and Laboratory departments. SOS hospital on the other hand was opened in 1985. It is also a mother and child hospital, located in Heliwa district in the south-eastern region of Mogadishu, about 12 kilometers from Banadir hospital. There are two departments at the hospital: Pediatric and Maternity sections. Both hospitals have Mother and child health centers (MCH). The MCH provides health care deliveries among mothers of reproductive age and children.Their services include immunizations. The study period was 14 months starting from October 2019 to December 2020.

We compared the routine childhood immunization coverage before the COVID-19 outbreak was reported in Somalia (October 2019, to February 2020), after the outbreak commenced with the pronouncement of the nationwide lockdown (March 2020 to August 2020) and after the suspension of the lockdown (September to December 2020). Since Somalia’s lockdown was 3 months, we used three months before and after lockdown to compare during lockdown. We collected monthly immunization report data from the register of the two target hospitals.

### Sampling and data collection procedure

The necessary data were obtained from the electronic database and manual EPI registration forms from SOS & Banadir hospitals, two medical students reviewed the immunization registers to collect the immunizations and documented the number of children who were immunized and the type of immunization they received. .

### Data management and analysis

Data were double-checked for completeness and kept in the locker for confidentiality. Once we got the data, we organized, arranged, and entered it in excel while ensuring the accuracy of the data. We subdivided variables into categories and rearranged them to obtain a good number of variables for analysis.

### Ethical approval

Ethical clearance was obtained from the Institutional Review Board domiciled at the Institute for Medical Research, SIMAD University. (Ref. No. 2022/IMRSU/FMHS/FR18/P030).

## Results

A total of 112,060 vaccinated children records were obtained across the two hospitals studied. The results showed that the number of vaccination visits before COVID-19 lockdown in SOS and Banadir hospital registries (October 2019 to February 2020) was 38,006 children, while 33,466 were obtained during first lockdown in Somalia ( March to August 2020), as well as 40,588 of the children were vaccinated post-lockdown (August 2020 to December 2020).

**Figure 1:**
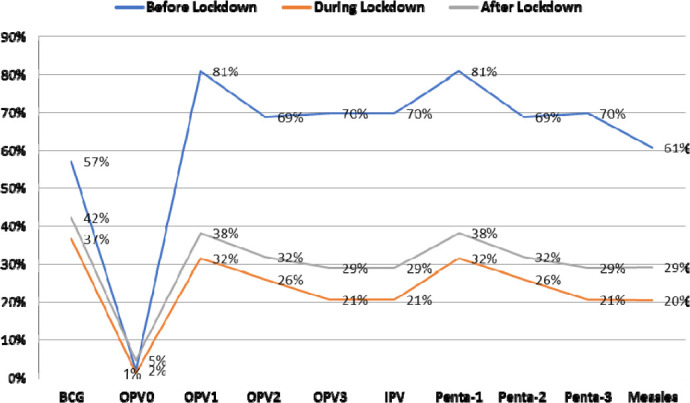
SOS Hospital EPI Coverage Trends Before, During and After Lockdown.

The above figure from SOS showed that almost all the vaccine types were disrupted during the COVID-19 lockdown. Penta-3 and Penta-1 had the highest drop with 49%, followed by Penta-2, 43%, and then measles with 41% with the least percentage coverage recorded for BCG with 20% (Figure 1).

**Figure 2:**
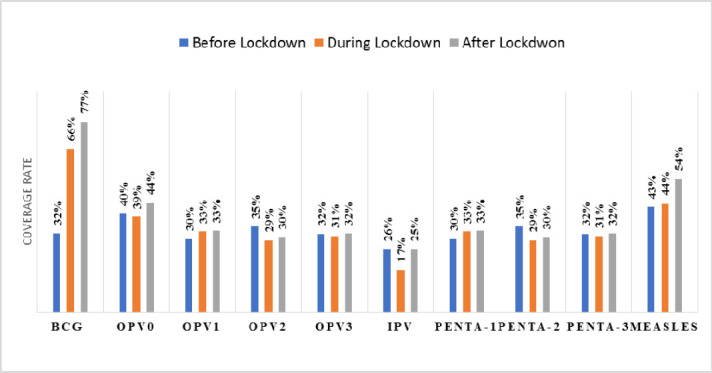
Banadir Hospital EPI Coverage Trends Before, During and After Lockdown.

Figure 2 showed vaccination coverage at Banadir Hospital before, during and after COVID19 lock down. Almost all variety vaccine types had nearly approximated each other. Penta 3 maximum dropped by 9% then followed penta 2 dropped by 6%. while BCG before COVID19 Lock down there was stock out at Banadir Hospital.

**Figure 3:**
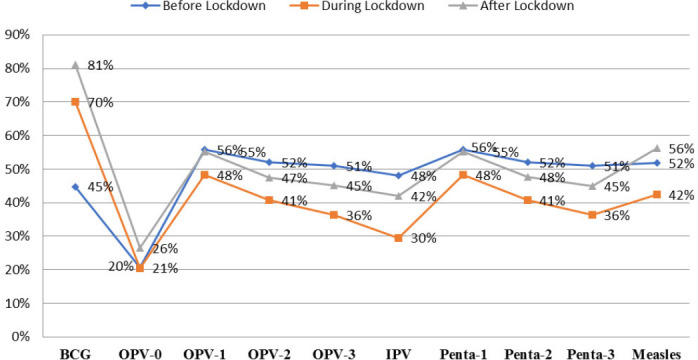
Combined data on EPI coverage trends at Banadir and SOS Hospitals.

Figure 3, on the other hand, showed a comparison of the EPI vaccine coverage rate where BCG coverage was found to increase by 25% during COVID-19 (Figure 3). However, it is important to note that BCG is administered immediately after birth and lockdown may not affect its administration. But regarding the other vaccines, there is noticeable drop rate, the extreme drop rate occurred by Penta-3. Penat3 dropped by 15% during lockdown (36%) compared before lockdown (51%) , followed by Penta-2 with 11%, measles by 10%, and lastly 8% Penta 1.

## Discussion

Previous studies have shown that response to a pandemic usually affects routine immunization as demonstrated in the British Columbia during the 2009 A/H1N1 influenza pandemic (Naus, 2021). This investigation attempts to determine the magnitude of the impact the ongoing COVID-19 pandemic has had on routine childhood immunization coverage in Mogadishu, Somalia, at the same time highlighting some of the salient factors that have played a major role in the attainment of the targeted coverage.

The WHO have reported that a total of 23 million children predominantly from war and conflict-ravaged countries have missed out on basic vaccines through routine immunization services in 2020. This figure indicates an increase of 3.7 million more than the number of children that missed these immunizations in the year 2019 (UNICEF, 2021). Gleaned from a similar WHO report on Somalia, in the year 2020, the government of Somalia set out to vaccinate 620,000 children against childhood tuberculosis, polio, diphtheria, pertussis, tetanus, hepatitis B, Haemophilus influenzae type B and measles. Unfortunately, that target was not achieved due to the COVID-19 pandemic (WHO-EMRO, 2020).

The findings of this study demonstrated that the number of children vaccination visits had significantly reduced since the COVID-19 outbreak. A major drop in vaccination visits was recorded between March and June 2020. This observation coincided with data gathered by the WHO and UNICEF Somalia country offices which showed that childhood immunization rates plummeted from March to May 2020, with a slight increase during the month of June (WHO-EMRO, 2020). This is thought to be a result of the movement restrictions put in place by the Somali government in its efforts to stem the tide of disease transmission. A similar trend of reduced coverage was also observed in November 2019, prior to the commencement of the lockdown. These findings conform with many previous reports where a reduction in the vaccination coverage and decline in the total number of vaccines administered was recorded following the COVID-19 outbreak (Lassi et al., 2021; Causeyid & Mosserid, 2022;; Shet et al., 2022). Also, the vaccination coverage rates for all the vaccine types dropped significantly, with Penta-3 being the vaccine with the highest decline with 27%, followed by Penta-2 at 11%, and then measles with 10%.

On April 24, 2013, the Somali authorities launched the incorporation of the new five-in-one-vaccine popularly called Pentavalent vaccine, which is a combination of five vaccines administered as one for protection against some of the highly prevalent illnesses among children which include, diphtheria, tetanus, whooping cough, Hepatitis B and Haemophilus influenzae type B which is responsible for several illnesses including meningitis, pneumonia among others (UNICEF 2013). The new vaccination policy was instituted to improve the health of the children and enhance the ranking of Somalia after being adjudged the country with the lowest immunization rates in the world. These disruptions in vaccine uptake are cause for concern as it may provide a window for other vaccine-preventable disease to emerge with greater morbidity and mortality. This danger is especially high in Somalia considering its vulnerability due to inadequate healthcare service and the fragility of the healthcare system. Similarly, the decline noted in this study for measles is a worrying sign because the disease is highly prevalent in most parts of Somalia, affecting children of all ages all year round. Prior to the COVID-19 outbreak, there was not enough management of the routine vaccination program owing to the country’s insecurity problem and poor health system. Unfortunately, the pandemic seems to have exacerbated the situation, thereby putting susceptible people and especially children in grave danger.

On a rather light note, the birth vaccines (BCG and Polio 0) were not considerably affected by the COVID-19 pandemic according to this study. This finding is like other reports elsewhere that showed little reduction or no reduction in birth vaccines (BCG, OPV-0) (Jain *et al.*, 2021; Mansour *et al.*, 2021). The minimal decline in birth vaccine uptake may be because of birth vaccinations traditionally administered at the hospital within 24 hours of giving birth, and the minimal impact of the COVID-19 lockdown on its uptake.

## Conclusion

The present investigation showed that COVID-19 pandemic had a significant effect on routine childhood vaccinations in Somalia. This study provides compelling evidence of a precipitous drop in routine childhood immunization since the inception of the outbreak till now. To stem the backsliding of childhood vaccination coverage, and work towards a healthier Somalia, urgent actions are necessary to address catch-up vaccinations, to strengthen the healthcare delivery systems to be able to sustain these routine immunizations. The results of this study may serve as a framework upon which strategies to improve coverage of the routine childhood immunization program during natural disasters can be built. We conclude by suggesting that routine childhood immunizations are prioritized, and strategies focused on achieving a significant and sustainable increase in immunizations during catastrophes. We also recommend further studies with a wider coverage in order to understand the magnitude of the COVID-19 impact on childhood immunization in Somalia.

### Limitations of this study

- We were not able to assess other factors that could have reduced the immunization coverage in Somalia.
- Since we were doing a retrospective study from the immunization register, we did not get the details of the immunized children such as the age and gender.

### Conflict of Interest

The authors declare that they had no conflict of interest associated with this study.

**Abbreviations:**
WHO-: World Health Organisation
UNICEF-: United Nations International Children Emergency Fund
BCG-: Bacille Calmette-Guérin
OPV-: Oral Polio Vaccine
